# Synovial hemangioma presenting with chronic painful infrapatellar mass: a case report

**DOI:** 10.1186/s12891-024-07708-3

**Published:** 2024-07-25

**Authors:** Wasaphon Suphakitchanusan, Napatr Wongboonkuakul, Kancharos Trakulsujaritchok, Chomkwan Decha-umphai, Thanapon Chobpenthai

**Affiliations:** 1 Department of Orthopaedics, Chulabhorn Hospital, Chulabhorn Royal Academy, 906 Kampangpetch 6 Street, Lak Si, Bangkok, Thailand; 2 Princess Srisavangavadhana College of Medicine, Chulabhorn Royal Academy, 906 Kampangpetch 6 Street, Lak Si, Bangkok, Thailand

**Keywords:** Synovial hemangiomas, Vascular abnormalities, Chondral damage, Arthroscopic excision, Knee pain, Palpable mass, Arthrocentesis, Minimally invasive procedure

## Abstract

**Background:**

Synovial hemangiomas are rare benign vascular anomalies surrounded by a synovial lining and were first described by Bouchut in 1856. These neoplasms can develop in the intra-articular region, resulting in effusions and knee pain. However, their cause remains unknown. Prompt diagnosis and intervention are critical to prevent chondral damage. Histopathological examination is used to achieve the diagnosis, which is often delayed because of a lack of specific clinical signs. This report describes a unique case in which a painful infrapatellar mass was diagnosed as a synovial hemangioma. The absence of typical magnetic resonance imaging (MRI) findings highlights the importance of arthroscopic excision for diagnosis and symptom relief.

**Case presentation:**

A 20-year-old woman presented with persistent anterior left knee pain that became exacerbated when she climbed stairs. Despite previous pain management and physical therapy, she developed a painful lump beneath her patella that worsened over time. She had also undergone arthrocentesis, but this did not relieve her pain. Physical examination revealed a palpable, immobile 5-cm mass along the patellar tendon with limited knee flexion and extension and normal ligament stability. T1-weighted fat-saturated MRI of the left knee with gadolinium-based contrast revealed a lobulated intra-articular mass in Hoffa’s fat pad that resembled a soft tissue chondroma. A biopsy of the mass was performed to provide histopathological evidence, confirming the benign nature of the mass. The subsequent excisional arthroscopy, combined with incision enlargement for mass removal, confirmed the histopathologic diagnosis of synovial hemangioma based on the presence of numerous dilated blood vessels and venous proliferation within sections of the synovium. Recovery was complete, and no residual tumor was detected on follow-up MRI after 1 year.

**Conclusion:**

This case study emphasizes the importance of arthroscopic excision over open surgery for patients with synovial hemangioma. The minimally invasive nature of arthroscopy combined with the well-encapsulated nature and location of the mass facilitates complete resection.

## Background

Synovial hemangiomas are benign vascular abnormalities that may originate in any anatomical structure surrounded by a synovial lining [1]. Synovial hemangiomas emerge from the mesenchymal layer underneath the synovial membrane and contain a variety of tissues such as adipose, fibrous, muscle, and thrombotic components within vessels. The cause of synovial hemangiomas remains uncertain, although they may arise in intra-articular areas, bursal spaces, or tendon sheaths [2]. Synovial hemangiomas account for less than 1% of all hemangiomas and cause chronic or recurring knee effusions and pain [1]. This uncommon condition is often misinterpreted in the clinical setting, and a preoperative diagnosis is estimated to be established in only 22% of cases; additionally, the diagnosis is sometimes delayed. Because of the lack of identifiable clinical signs and the existence of intra-articular malignancies, synovial hemangiomas are commonly misdiagnosed and only verified after histological examination [3]. A lobulated intra-articular mass with distinctive signal intensity on magnetic resonance imaging (MRI) is often indicative of a synovial hemangioma [4]. Delayed therapy has the potential to cause chondral damage and subsequent degeneration [5]. We herein report a case involving a 20-year-old woman who presented with persistent unilateral knee discomfort. This report complies with the SCARE criteria and PROCESS guidelines [6, 7].

## Case presentation

A 20-year-old woman presented with a 5-year history of chronic knee pain. She was healthy and had no history of trauma. The pain was limited to the anterior region of her left knee. It was exacerbated by activities, particularly those involving stairs, and did not improve with any medications or physical therapy. During this time, she noticed a small but painful lump below her patella. She had developed swelling in her left knee accompanied by increased pain, prompting her to undergo arthrocentesis at a different medical facility. Despite arthrocentesis, the pain became increasingly severe, and the mass grew in size. Thus, she visited Chulabhorn Hospital, a tertiary-level, academic-based medical center.

On physical examination, a palpable mass was identified along the patellar tendon. The mass was soft, tender, immobile, and approximately 5 cm in diameter. Her left knee was slightly swollen, exhibiting limited terminal flexion and extension and a range of motion of approximately 10 degrees. Ligament stability test results were within the normal ranges, and Hoffa’s test revealed impingement of the infrapatellar fat pad. All other physical examination findings were unremarkable.

**Fig. 1:**
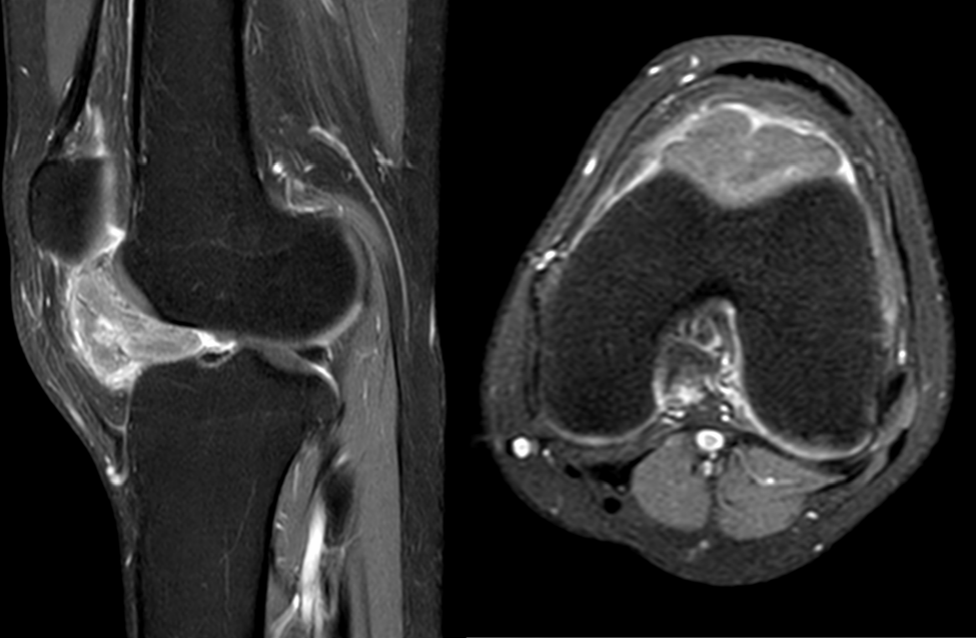
Sagittal (left) and axial (right) post IV with paramagnetic contrast medium, T1-weighted, with fat suppression, magnetic resonance images showed a lobulated intra-articular mass within Hoffa’s fat pad. The mass exhibited mild heterogeneous contrast uptake

Given the soft tissue abnormalities, the patient was referred for further evaluation by MRI [5]. The MRI examination showed a lobulated intra-articular mass within Hoffa’s fat pad (Fig. 1). This mass showed iso signal intensity on T1-weighted images and intermediate to high signal intensity on T2-weighted images, with some areas showing low signal intensity on gradient echo (GE) sequences. Furthermore, a peripheral hypointense rim was well defined on T2-weighted and GE sequences and showed moderate and heterogeneous enhancement. The mass measured 3 × 1 × 3 cm. There was no indication of chondral injury or involvement. The patient had normal patellar alignment and appropriate joint effusion. The knee ligaments, medial meniscus, lateral meniscus, and neurovascular bundles lacked the ability to stretch. As a result, the mass was suspected to be a soft tissue chondroma of the Hoffa fat pad. Therefore, localized nodular synovitis of the infrapatellar fat pad and synovial hemangioma were two differential diagnoses [8].

The patient underwent a tissue biopsy in the outpatient department under local anesthesia with 2% lidocaine and no adrenaline, and she received intravenous analgesia with 30 mg ketorolac. The pathology report was nonspecific, revealing only fat tissues. Given the preliminary diagnosis of a soft tissue chondroma based on the MRI and biopsy results, the surgical orthopedics team recommended excision. The goal was to alleviate the symptoms and confirm the diagnosis. An arthroscopic approach was preferred over open surgery for several reasons, including the minimally invasive nature of arthroscopy; the location and well-encapsulated nature of the mass, which facilitated complete resection using an arthroscope; and the desire for optimal cosmesis.

**Fig. 2:**
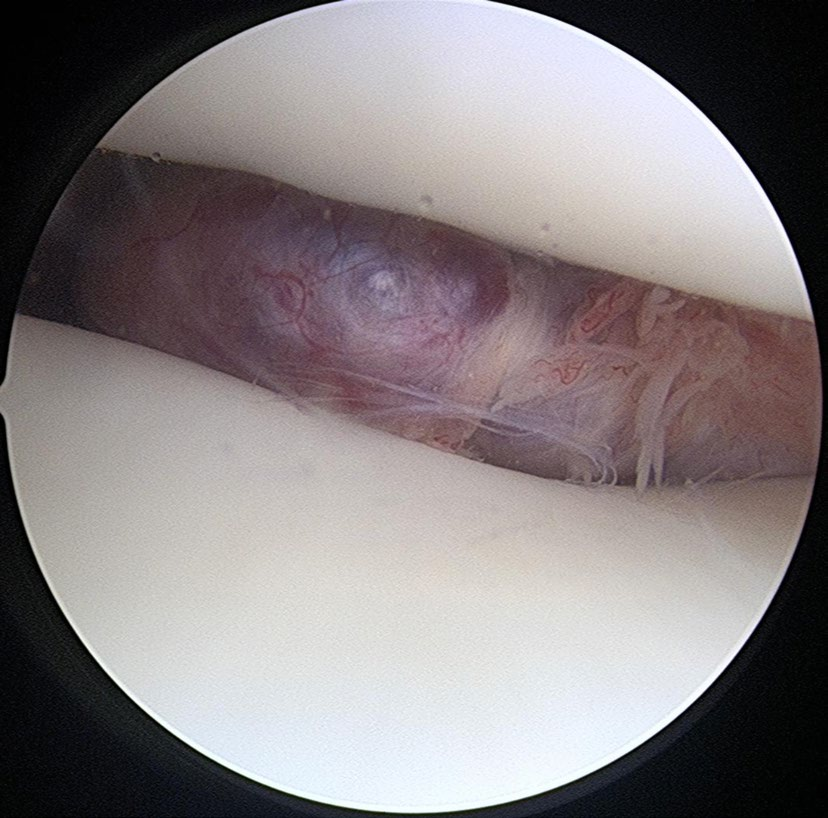
Arthroscopic view from the suprapatellar pouch revealed an abnormal mass between the patella and the trochlear groove

One month later, the patient underwent arthroscopic excision of the left knee mass. A spinal block and an adductor canal block were used for local anesthesia during the operation. The patient lay supine on the operating table, with the operated leg hanging freely off the edge. Anterolateral and anteromedial portals were strategically placed approximately 1.5 cm from the patellar tendon. These placements were slightly lateral and medial in relation to standard portals. This modification was informed by the MRI results to maintain a safe distance from the mass and avoid intrusion. Because the mass was located in the anterior compartment, the superolateral portal was designated as the viewing portal (Fig. 2). The superomedial portal was concurrently used to manipulate the mass in conjunction with the anterolateral and anteromedial portals. The well-developed capsule of the mass required blunt dissection to distinguish it from adjacent tissue. Following successful isolation, the mass was extracted en bloc via an extended incision at the anteromedial portal.

**Fig. 3:**
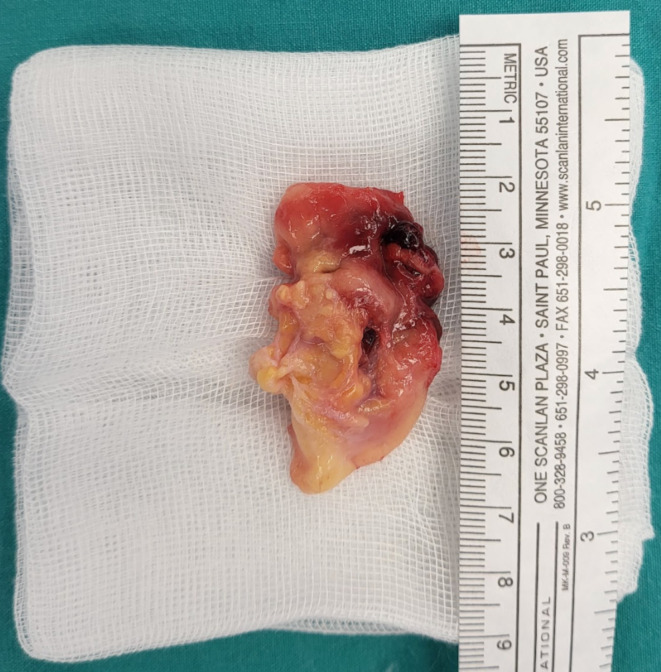
The gross specimen exhibited irregular fatty tissue fragments and measured 4.5 × 2.5 × 2.0 cm

**Fig. 4:**
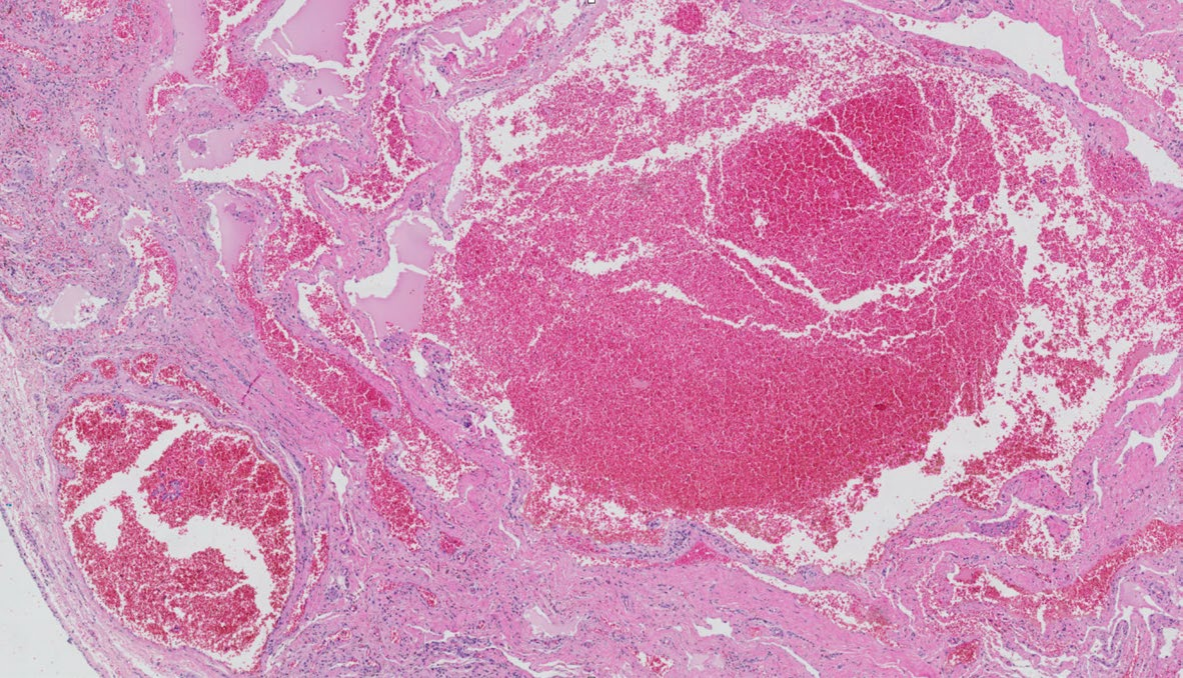
Histopathology (scale, 200×; hematoxylin and eosin stain) showed synovial segments with numerous dilated blood vessels and evidence of venous proliferation

The gross specimen consisted of irregular fatty tissue fragments and measured 4.5 × 2.5 × 2.0 cm (Fig. 3). Microscopic examination revealed numerous dilated blood vessels and evidence of venous proliferation in segments of the synovium (Fig. 4). A few areas showed indications of past hemorrhage with hemosiderin-laden macrophage deposits. A piece of bone was also visible within the stromal tissue. The remaining tissue consisted of a few infiltrating lymphocytes and plasma cell clusters. The pathologist’s report concluded that the resection margin was negative and that the resected mass was a synovial hemangioma.

Postoperative exercises were immediately initiated to improve the range of motion and patellar mobilization and therefore prevent adhesions. The patient’s recovery progressed well, and she was instructed to progress to full weight bearing as tolerated. At 2 months postoperatively, she had resumed her daily activities and started working without pain. A follow-up MRI examination at 6 months confirmed that the mass had been completely eliminated, and no residual tumor was visible. One year postoperatively, the patient reported a visual analogue scale score of 1 out of 10, indicating minimal pain. Furthermore, her International Knee Documentation Committee score was 95, indicating a positive surgical outcome and rehabilitative progress.

## Discussion and conclusions

Intra-articular tumors encompass a broad spectrum of pathologies, including benign and malignant lesions. Differential diagnoses for an intra-articular mass include synovial hemangioma, soft tissue chondroma, PVNS, ganglion cyst, lipoma arborescens, and synovial sarcoma [9]. Performing a biopsy in the presence of a large intra-articular mass is crucial for accurate diagnosis, guiding treatment, avoiding unnecessary surgery, and assessing prognosis [10]. Ultrasound-guided biopsy offers a precise, minimally invasive, and cost-effective method for obtaining diagnostic tissue samples, ensuring accurate targeting of the lesion and reducing complications. This case underscores the importance of considering a wide differential diagnosis and the role of biopsy in confirming the nature of intra-articular masses for optimal patient management [9].

The present case of a synovial hemangioma is distinct in that it presented as a palpable knee mass rather than the more common symptom of knee discomfort and effusion. Additionally, the mass was located in the infrapatellar region rather than in the suprapatellar pouch of the knee, which is the most common location of synovial hemangiomas [1, 11].

When a synovial hemangioma is suspected, imaging is first performed to rule out other possibilities. The surgical plan is then established. MRI features that indicate a synovial hemangioma include high signal intensity on T2-weighted imaging, low signal intensity on T1-weighted imaging, a lobulated lesion with well-defined margins, and signal intensity brighter than fat with linear low-signal structures throughout the lesion. Synovial hemangiomas also show a substantial rise in the signal intensity in post-contrast sequences [4, 12].

In the present case, MRI revealed areas with poor signal strength in the GE sequence. However, it did not show the linear low-signal structures that are typically found in synovial hemangiomas. As a result, the appearance was similar to a soft tissue chondroma in Hoffa’s fat pad [8]. The lack of specific imaging findings and biopsy results highlight the importance of collecting a pathology sample for a definitive diagnosis. The surgical team in our case decided on excision, demonstrating the difficulties of identifying the condition. Synovial hemangiomas have a high rate of recurrence after arthroscopic excision [13]. However, the decision to perform arthroscopic excision was based on its minimally invasive nature, the position of the mass inside the limited space of the infrapatellar region, and the desire for an optimal cosmetic outcome. Furthermore, the preoperative evaluation demonstrated a well-encapsulated mass; the surgical team thus concluded that it was likely to be entirely removed, reducing the probability of recurrence.

Comparing arthroscopic and open excision methods for intra-articular masses reveals distinct advantages and limitations. Arthroscopic excision offers several benefits, including being minimally invasive and having better cosmetic outcomes, it may be limited by technical demands and the risk of incomplete resection for larger or more complex masses. Bawa AS et al. report highlights a long-term follow-up of a patient with synovial hemangioma treated with arthroscopic synovectomy, patient reports that two years after surgery, with a painless knee and a full range of movement [5]. Auran et al. emphasized the benefits of arthroscopic excision for smaller, well-defined masses, recommending open excision for larger or more complex lesions due to better access and resection capabilities [1]. Uemura et al. reported excellent outcomes with minimal complications and quick recovery following arthroscopic resection of a knee joint synovial hemangioma but emphasized the need for surgical expertise [3]. Li et al. found that while arthroscopy offered better cosmetic results and quicker recovery, open excision was more reliable for complete resection of larger masses [13]. Open excision provides direct access and reduces the risk of incomplete resection but is associated with higher morbidity and cosmetic concerns. The choice of technique should be individualized based on the specific characteristics of the mass and the patient’s overall condition.

In summary, synovial hemangiomas are uncommon benign vascular anomalies that can appear in a variety of anatomical locations, including intra-articular areas. Delayed diagnosis can result in chondral damage and subsequent degeneration. This case report describes a synovial hemangioma in the infrapatellar region that presented as a palpable mass rather than an effusion. The diagnosis was challenging because of aberrant MRI features and vague biopsy results, and mass excision was required for both diagnosis and symptom alleviation. Despite the possibility of recurrence, a minimally invasive technique was chosen, and the patient recovered completely with reduced pain and no tumor recurrence. These findings underscore the need for early diagnosis and personalized care in patients with synovial hemangiomas.

## Data Availability

All the data are available from the corresponding author upon reasonable request.

## Abbreviations

MRI: Magnetic resonance imaging
GE: Gradient echo

## References

[1] Auran RL, Martin JR, Duran MD, de Comas AM, Jacofsky DJ. Evaluation and management of intra-articular tumors of the knee. J Knee Surg. 2022;35:597–606. 10.1055/s-0042-1743223. 35189664 10.1055/s-0042-1743223

[2] Kolluru AR, Gautam AA, Garg S, Tamboli AI. Magnetic resonance imaging of synovial diseases of knee. J Datta Meghe Inst Med Sci Univ. 2023;18:291–8. 10.4103/jdmimsu.jdmimsu_29_23. 10.4103/jdmimsu.jdmimsu_29_23

[3] Uemura R, Kumagai K, Kubo M, Mimura T, Yayama T, Imai S. Knee joint synovial hemangioma treated with arthroscopic resection without hemarthrosis: a case report. Int J Surg Case Rep. 2024;116:109352. 10.1016/j.ijscr.2024.109352. 38320414 10.1016/j.ijscr.2024.109352 PMC10850950

[4] Sheldon PJ, Forrester DM, Learch TJ. Imaging of intraarticular masses. Radiographics. 2005;25:105–19. 10.1148/rg.251045050. 15653590 10.1148/rg.251045050

[5] Bawa AS, Garg R, Bhatnagar K, Singal S. Synovial hemangioma of the knee management and excellent outcome 2 years after arthroscopic synovectomy in a 25-year-old male with a 20-year history. J Orthop Case Rep. 2017;7:17. 10.13107/jocr.2250-0685.786. 29051872 10.13107/jocr.2250-0685.786 PMC5635178

[6] Sohrabi C, Mathew G, Maria N, Kerwan A, Franchi T, Agha RA. The SCARE 2023 guideline: updating consensus Surgical CAse REport (SCARE) guidelines. Int J Surg. 2023;109:1136–40. 10.1097/JS9.0000000000000373. 37013953 10.1097/JS9.0000000000000373 PMC10389401

[7] Agha RA, Sohrabi C, Mathew G, Franchi T, Kerwan A, O’Neill N, et al. The PROCESS 2020 guideline: updating consensus Preferred reporting of CasE series in surgery (PROCESS) guidelines. Int J Surg. 2020;84:231–5. 10.1016/j.ijsu.2020.11.005. 33189880 10.1016/j.ijsu.2020.11.005

[8] Helpert C, Davies AM, Evans N, Grimer RJ. Differential diagnosis of tumours and tumour-like lesions of the infrapatellar (Hoffa’s) fat pad: pictorial review with an emphasis on MR imaging. Eur Radiol. 2004;14:2337–46. 10.1007/s00330-004-2491-1. 15449005 10.1007/s00330-004-2491-1

[9] Sheldon PJ, Forrester DM, Learch TJ. Imaging of intraarticular masses. Radiographics. 2005;25(1):105–19. 15653590 10.1148/rg.251045050

[10] Auran RL, Martin JR, Duran MD, de Comas AM, Jacofsky DJ. Evaluation and management of intra-articular tumors of the knee. J Knee Surg. 2022;35(6):597–606. 35189664 10.1055/s-0042-1743223

[11] Beltrame V, Romanucci G, Zulian F, Stramare R. Synovial hemangioma of infrapatellar (Hoffa) fat pad: magnetic resonance imaging and ultrasound features. J Pediatr. 2016;172:222–3. 10.1016/j.jpeds.2016.01.052. 26922106 10.1016/j.jpeds.2016.01.052

[12] Larbi A, Viala P, Cyteval C, Snene F, Greffier J, Faruch M, et al. Imaging of tumors and tumor-like lesions of the knee. Diagn Interv Imaging. 2016;97:767–77. 10.1016/j.diii.2016.06.004. 27397886 10.1016/j.diii.2016.06.004

[13] Li W, Yu F, Wu X, Jiang CQ, You T, Zhong QW, et al. Extra-articular arthroscopic excision of the intramuscular hemangioma in a lower extremity has better therapeutic effect and higher satisfaction with appearance comparing with open excision. Indian J Surg. 2021;83:974–9. 10.1007/s12262-020-02566-4. 10.1007/s12262-020-02566-4

